# Mitophagy and Bip–PERK–eIF2α–ATF4 Axis‐Mediated ER Stress Mediate Miriplatin‐Loaded Liposome's Anti‐Colorectal Cancer Action

**DOI:** 10.1111/cpr.70153

**Published:** 2025-12-19

**Authors:** Cong Zhao, Yuhan Qiu, Xiaowei Wang, Mengyan Wang, Li Liu, Xiaojun Zhao, Zixiang Gao, Rongguang Shao, Guimin Xia, Wuli Zhao

**Affiliations:** ^1^ State Key Laboratory of Respiratory Health and Multimorbidity, Key Laboratory of Antibiotic Bioengineering, Ministry of Health, Laboratory of Oncology Institute of Medicinal Biotechnology, Chinese Academy of Medical Sciences and Peking Union Medical College Beijing China; ^2^ Department of Pharmacy Zhujiang Hospital, Southern Medical University Guangzhou Guangdong China

## Abstract

LMPt enters the cell mainly through caveolin‐mediated endocytosis, and then fuses with endosomes and lysosomes to deliver MPt to mitochondria and the endoplasmic reticulum to induce mitophagy based on the fusion of lysosomes and mitochondria, and endoplasmic reticulum stress and subsequent apoptosis via the Bip–PERK–eIF2α–ATF4 axis to exert an anti‐breast cancer effect.

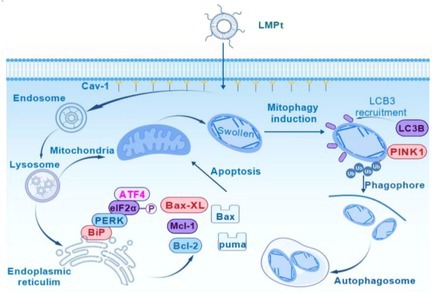

To the Editor,

Colorectal cancer is a common gastrointestinal cancer. It ranks third among high‐incidence tumour types (10.0%) and second among tumour types with low survival rates (9.4%) [1]. Chemotherapy is the primary treatment for colorectal cancer, and oxaliplatin (OXA) is recommended as a first‐line therapy [2]. Although oxaliplatin exhibits prominent anti‐colorectal cancer efficacy, the lack of in vivo distribution specificity, neurotoxicity and myelosuppressive toxicity significantly limit its clinical application. Similarly, other chemotherapeutics also face analogous challenges. Therefore, safer and more effective agents are needed to enhance the therapeutic outcomes of colorectal cancer. Liposomes acting as the most effective drug delivery systems have been used to improve the toxicity and side effects of various chemotherapeutic drugs [3]. Furthermore, liposomes selectively aggregate in specific parts of the body, enhancing permeability and accumulating at tumour sites, which is a kind of passive targeting effect of liposomes.

Miriplatin (MPt), a third‐generation platinum drug, is administered as an MPt/lipiodol suspension via intrahepatic arterial injection for treating unresectable hepatocellular carcinoma (HCC) due to its poor solubility [4]. MPt contains two long myristoyl chains, is highly lipophilic, and is structurally similar to phospholipids, forming phospholipid bilayers in liposomes. Based on the structural similarity, our team prepared a novel liposomal formulation of MPt, also named lipomiriplatin (LMPt) (Figure S1A), which retained the good anti‐tumour activity of MPt while reducing the toxic side effects.

In this study, we focus on exploring the mechanism of LMPt on colorectal cancer, including the endocytosis pathway, subcellular location and targeted organelles after entering into cells, the induced pathology and molecular pathways leading to proliferation inhibition. Our study demonstrated the potent anti‐colorectal effect of LMPt with other anti‐cancer assays from previous research. We found that LMPt mainly entered cells through the caveolin‐mediated endocytosis pathway. It was first localised to endosomes, and it caused endoplasmic reticulum stress and induced apoptosis. Subsequently, it was transported into mitochondria to induce mitophagy, an entirely different anti‐cancer mechanism from the previously reported platinum agents.

## 1 Results

### 1.1 Superior Anti‐Colorectal Cancer Activity Is Observed in LMPt‐Treated Colorectal Cancer

We detected sole MPt's effect on colorectal cancer proliferation and found that at the highest solubility dose of 3.4 μM, it displayed almost no anti‐cancer effect at a style of MPt itself (Figure S1B). Our previous study showed that the anti‐colorectal cancer effect of LMPt in vitro and in vivo, and these effects were superior to those of oxaliplatin [5]. In this report, we further demonstrated LMPt's activity with different assays, colorectal cancer cells and doses of LMPt. Results showed that there was an obvious dose‐dependent (Figure S1C) and time‐dependent (Figure S1D) effect on colorectal cancer cells HCT8 and HT29. Meanwhile, with increasing doses of LMPt, we also observed that the shape of treated cells gradually became spindle‐shaped and shrank. Additionally, vacuoles appeared at lower doses, and their numbers progressively increased at higher doses (Figure S1E). EdU is a thymine nucleoside analogue that replaces thymine in the process of cell proliferation and incorporates the DNA molecule that is replicating, indicating the proliferative ability of cells based on the specific reaction of EdU with Apollo‐like fluorescent dyes. We used the EdU assay to examine the effect of LMPt on DNA replication in colorectal cancer cells, and the results showed that the positive rate of EdU in LMPt‐treated colorectal cancer cells was significantly reduced compared with the control group, and the effect of LMPt on cell proliferation inhibition was dose‐dependent (Figure S1F,G).

Cell clone formation assay is an important method to evaluate cell proliferation and cell sensitivity to drugs. We treated colorectal cancer cells HCT8 and HT29 with 0.5, 1 and 2 μM LMPt, respectively, for 7 days, and when the cells formed obvious clones, they were photographed and the number of cell clones was counted. The results showed that the LMPt could significantly inhibit the proliferation of colorectal cancer cells, and the inhibitory effect was concentration‐dependent (Figure S1H,I).

### 1.2 Caveolin‐Dependent Endocytic Pathway Mediates LMPt's Cellular Entry in Colorectal Cancer Cells

**FIGURE 1:**
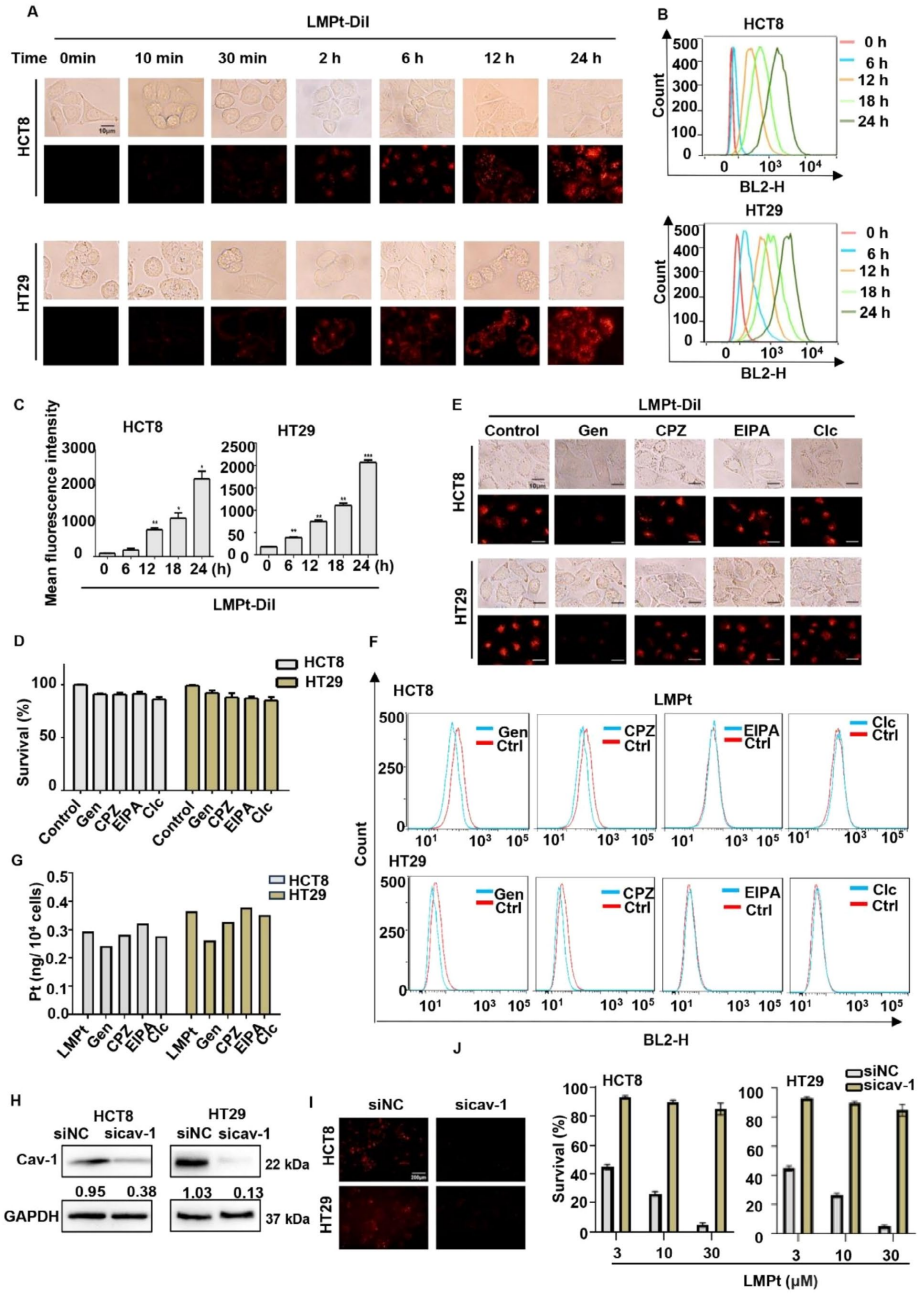
Caveolin‐dependent endocytic pathway mediates LMPt's cellular entry in colorectal cancer cells. HCT8 and HT29 cells were treated with 5 μM DiI‐labelled LMPt for indicated time, and the intracellular uptake of LMPt was detected with fluorescence microscope (A) and flow cytometry (B). Scale bar, 10 μm. The data were processed by the FlowJo software. *p < 0.05, **p < 0.01, ***p < 0.001, compared with mean fluorescence intensity at 0 min. (C) Quantitative analysis of (B). (D) HCT8 and HT29 cells were treated with diverse endocytosis inhibitors for 12 h, and then MTT assay was used to detect the effect of cell viability. The data were expressed as mean ± SEM. HCT8 and HT29 cells were pre‐treated with different endocytosis inhibitors for 2 h, and then treated with LMPt for 24 h, the fluorescence microscope (E) and flow cytometry (F) were used to detect the effect of endocytosis inhibitors on intracellular fluorescence intensity, and Pt content was measured with ICP–MS (G). Si‐caveolin‐1 was transfected into cells and knockout efficiency was detected by Western blot (H) and intracellular uptake of LMPt was detected with fluorescence microscopy (I). Scale bar, 200 μm. (J) Si‐caveolin‐1 was transfected into HCT8 and HT29 cells for 24 h, and cell viability was detected after treatment with LMPt at concentrations of 3, 10, and 30 μM for 48 h.

The ability of tumour cells to uptake drugs is critical for drug activity. In order to observe the uptake of LMPt, the red fluorescent probe Dil was used to encapsulate LMPt to analyse the location of LMPt. At 10, 30 min, 2, 6, 12 and 24 h after LMPt treatment in HCT8 and HT29 cells, the uptake of the LMPt was evaluated with a fluorescence microscope. Results showed that the liposomes gathered around the cell membrane at 30 min and began to enter the cell, and the intracellular fluorescence intensity increased with the extension of time, and the cell entry was basically complete at 12 h (Figure 1A). Next, we used flow cytometry to quantitatively measure the fluorescence intensity of cells after ingestion of LMPt. Results showed that the average intracellular fluorescence intensity gradually increased with the prolongation of culture time (Figure 1B,C), indicating that there was a time‐dependent uptake of LMPt in colorectal cancer cells.

Generally, most nanoparticles enter the cell through endocytosis [6], which mainly includes the following types: pinocytosis and phagocytosis. Pinocytosis includes caveolin‐dependent endocytosis, clathrin‐dependent endocytosis [7], non‐clathrin and caveolin‐dependent endocytosis [8] and macropinocytosis [9]. In order to explore the endocytic pathway of LMPt, we used chlorpromazine (CPZ) as an inhibitor of clathrin‐dependent endocytosis, genistein (Gen) as an inhibitor of caveolin‐dependent endocytosis, amiloride (EIPA) as an inhibitor of macropinocytosis, and colchicine (Clc) as an inhibitor of non‐clathrin and caveolin‐dependent endocytosis, respectively, to explore the effects of endocytosis inhibitors on LMPt entry into cells.

First, we evaluate the toxicity of the inhibitors used. Colorectal cancer cells HCT8 and HT29 were treated with 10 μM genistein, 10 μM amiloride, 5 μg/mL chlorpromazine and 0.1 μg/mL colchicine as previous reports, respectively, and cell viability was detected by MTT assay. The results showed that the survival of cells was not affected by inhibitors (Figure 1D), indicating that the above concentrations of endocytic inhibitors were acceptable for further assays. Then, in HCT8 and HT29 cells, we added the above concentrations of inhibitors for 2 h prior to exposure to Dil‐labelled LMPt to evaluate the cellular entry pathway of LMPt. Flow cytometry was used to quantitatively analyse the intracellular fluorescence intensity, and the results showed that the intracellular fluorescence content of HCT8 cells pretreated with genistein was reduced significantly (Figure 1E,F). In HT29 cells, we also got similar results, suggesting the caveolin‐dependent endocytic pathway involved in LMPt's cellular entry.

To further verify that caveolin‐mediated pathway was the key to LMPt's entry and subsequent anti‐cancer effect, we utilised endocytosis inhibitors to treat cells for 2 h prior to the addition of LMPt to detect intracellular platinum content by ICP–MS in HCT8 and HT29 cells, and the results showed that the intracellular platinum content treated with genistein was most significantly reduced compared with the control group, while the other inhibitors only exerted a slight effect on the inhibition of platinum uptake (Figure 1G).

Caveolin‐1 is reported as a fundamental protein involved in the caveolin‐dependent endocytic pathway [10]. To evaluate its potential role in LMPt entry into cells, we knocked down caveolin‐1 in HCT8 and HT29 cells and verified it by Western blot (Figure 1H). Then, in cells with caveolin‐1 silenced, DiI‐labelled LMPt was added for detection of LMPt entry. The results revealed that the cellular entry of LMPt was significantly inhibited after caveolin‐1 was knocked down compared to the control group (Figure 1I). Meanwhile, knocking out caveolin‐1 reversed the anti‐colorectal cancer effect of LMPt (Figure 1J). In summary, it can be concluded that the cellular uptake of LMPt is mainly dependent on caveolin 1‐mediated endocytosis.

### 1.3 LMPt Mainly Locates in Mitochondria and Endoplasmic Reticulum (ER) Followed by Preliminary Cellular Treatment

Usually, after entering into cells, liposomes would firstly enter into endosomes and subsequently into lysosomes for primary treatment prior to entering into targeting organelles. In order to investigate the transport of LMPt after entering into cells, we labelled the endosomes and lysosomes with fluorescent dyes and treated the colorectal cancer cells with Dil‐labelled LMPt. The results showed that LMPt began to localise to endosomes 2 h after exposure to LMPt (Figure S2A) and 6 h later it was found in lysosomes (Figure S2B), and subsequently, the accumulation of LMPt in endosomes and lysosomes was found to gradually increase. The above results suggested that LMPt firstly fused with the endosomes to form the primary endosomes after entering the cell, and then formed primary endosomes eventually via fusing with lysosomes [11] to process LMPt.

Then we continued to explore the targeting organelles of LMPt followed by treatment with LMPt. HCT8 and HT29 cells were treated with 30 μM LMPt for 24 h, and the vital organelles such as mitochondria, endoplasmic reticulum and genomic DNA components were extracted, respectively, and the platinum content was detected with ICP–MS. The results showed that LMPt was majorly distributed in mitochondria and endoplasmic reticulum, but less in genomic DNA (Figure S2C), suggesting that the mechanism of action of LMPt may be different from that of traditional platinum anti‐tumour drugs, and it does not exert anti‐tumour effects by cross‐linking with DNA to inhibit DNA replication.

Electron microscopy was introduced to evaluate whether LMPt‐locating organelles exhibited pathological changes although in them there were amounts of platinum accumulation. Images of electron microscopy showed that compared with the control, LMPt‐treated cells exhibited swollen mitochondria, and the mitochondrial cristae structure was broken and particles in the matrix were lessened, and vacuolar degeneration appeared. These results indicated that LMPt significantly damaged mitochondria, including their inner and outer structures. Correspondingly, we also observed some tiny vacuolates surrounded by membranes that were probably the damaged endoplasmic reticulum (Figure S2D). Based on the above findings, with different assays, we further ascertained the pathological changes of mitochondria and ER. MitoTracker Green FM was a fluorescence agent and was usually used to detect mitochondrial morphology. In our assay, after LMPt treatment and MitoTracker Green FM staining, we observed that mitochondria lost many original structures and the whole outline became half of untreated cells. The remaining structure also lost its original orderly and rod‐shaped structure and became concentrated and dotted (Figure S2E). ImageJ was used for quantitative analysis, and it was found that the mitochondria were broken, the spherical and dotted rod‐like structures appeared, and the mitochondrial footprint and branch length were reduced, which further indicated that LMPt induced mitochondrial damage (Figure S2F). Then we applied JC‐1 [12] to assess mitochondrial membrane potential representing mitochondrial function in response to LMPt. HCT8 and HT29 cells were treated with LMPt at concentrations of 0, 10 and 30 μM, respectively, and after 24 h, JC‐1 staining was incubated and observed under a fluorescence microscope. The results showed that compared with the control group, the red fluorescence intensity of the LMPt administration group was weakened, and the green fluorescence intensity was enhanced (Figure S2G), which was directly proportional to the administered concentration. The above results showed that LMPt could reduce the mitochondrial membrane potential of colorectal cancer cells and cause mitochondrial damage, and the degree of damage was positively correlated with the administration concentration.

Next, we transfected HCT8 cells with an ER–GFP plasmid labelled endoplasmic reticulum lumen and an ER‐Sec61 plasmid labelled endoplasmic reticulum membrane and established the stably expressed cell lines, labelling the endoplasmic reticulum cavity protein and endoplasmic reticulum membrane protein with green fluorescence. After 30 μM LMPt treatment for 24 h, the endoplasmic reticulum morphology in the cells was observed under fluorescence microscopy, and it was found that there were multiple vacuoles in the endoplasmic reticulum membrane after administration, and the endoplasmic reticulum cavity was damaged, indicating that LMPt caused endoplasmic reticulum damage in colorectal cancer cells (Figure S2H).

### 1.4 LMPt Induces Mitophagy and Endoplasmic Reticulum Stress‐Mediated Apoptosis

In order to study the mechanism of anti‐colorectal cancer of LMPt, we extracted RNA for sequencing analysis. KEGG pathway enrichment analysis indicated that there were 153 and 41 differential genes in the processes of cell metabolism and cell growth and death, respectively, and 123 differential genes were enriched in the autophagy pathway, 120 genes were enriched in the mitochondrial autophagy pathway, and nine differentially expressed genes were enriched in the apoptotic pathway (Figure S3A). This suggests that the mechanism of LMPt may be related to the mitophagy of colorectal cancer cells and apoptosis.

In order to further explore the mechanism of LMPt, we respectively used the autophagy inhibitor 3‐Methyladenine (3‐MA), the apoptosis inhibitor Z‐VAD, and the programmed necrosis inhibitor Necrostatin‐1 (Nec‐1) to treat colorectal cancer cells HT29 and HCT8 cells at a concentration of 40 μM together with 5 μM LMPt, and the MTT method was used to explore the effect of inhibitors on LMPt. Compared to the control group, the autophagy inhibitor 3‐MA treatment and the apoptosis inhibitor Z‐VAD all improved cell viability, restored the effect of LMPt (Figure S3B,C), and were consistent with the results of gene enrichment analysis, indicating that LMPt may inhibit cell proliferation by inducing apoptosis and autophagy in colorectal cancer cells.

During mitophagy occurrence, Microtubule‐associated protein light chain 3 (LC3) is conjugated to phosphatidylethanolamine to form LC3‐phosphatidylethanolamine conjugate (LC3‐II), which is recruited to the membrane of the autophagosome containing the damaged mitochondria [13]. Then the mitochondria would fuse with lysosomes to form mitochondrial autophagosomes to degrade the damaged mitochondria. Thus, firstly we detected whether the LC3‐II level increased in mitochondria and found that both in HCT8 cells and in HT29 cells, the expression level of LC3II in the mitochondria increased after administration (Figure S3E), indicating that the LMPt promoted the occurrence of mitophagy. Then we applied Lysotracker Green to label lysosomes and MitoTracker Red to label mitochondria to assess the co‐localisation of mitochondria and lysosomes in cells. The co‐localisation of lysosomes and mitochondria was observed under fluorescence microscopy after 24 h of 30 μM LMPt treatment (Figure S3D). Finally, we extracted mitochondria for ubiquitination evaluation, and the results also demonstrated that LMPt induced mitochondrial ubiquitination (Figure S3F). All these results showed that LMPt could induce mitochondria mitophagy via binding with lysosomes to subject the damaged mitochondria.

### 1.5 LMPt Induces Endoplasmic Reticulum Stress‐Mediated Mitochondrial Apoptosis via Bip–PERK–eIF2α–ATF4 Axis

**FIGURE 2:**
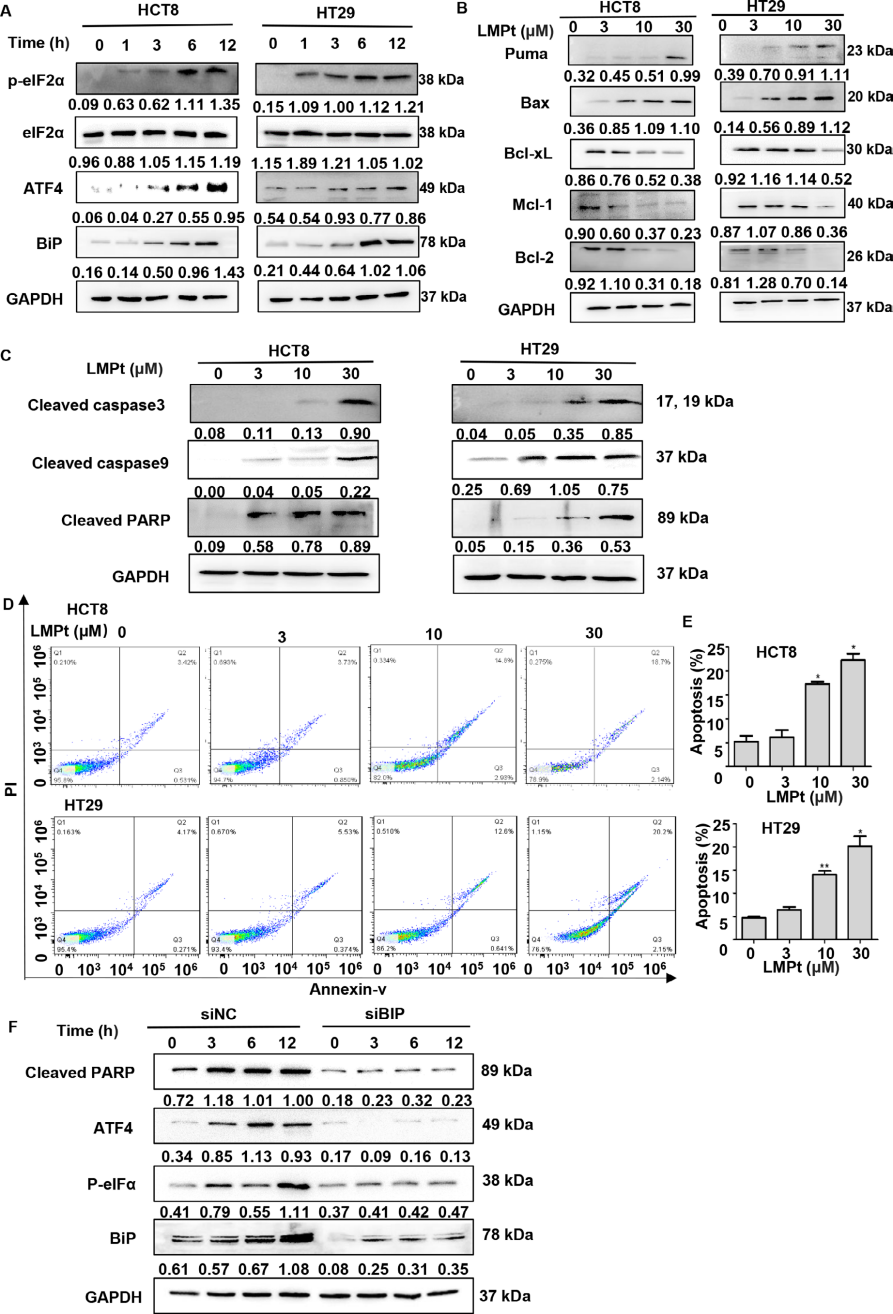
LMPt induces endoplasmic reticulum stress‐mediated mitochondrial apoptosis via the Bip–PERK–eIF2α–ATF4 axis. (A) HCT8 and HT29 cells were treated with 30 μM LMPt for indicated time for the detection of proteins in BiP–PERK–eIF2α–ATF4 axis with Western Blot. (B) HCT8 and HT29 cells were treated with 0, 3, 10 or 30 μM LMPt for 48 h. LMPt increased the expression levels of mitochondrial pro‐apoptosis related proteins in HCT8 and HT29 cells, while decreased the expression levels of anti‐apoptosis related proteins. (C) HCT8 and HT29 cells were treated with 0, 3, 10 or 30 μM LMPt for 48 h, and apoptosis‐associated proteins were detected by Western blot. (D) HCT8 (the upper half part) and HT29 (the lower half part) cells were treated with different concentrations of LMPt for 24 h, then cells were stained with propidium iodide and Annexin V‐FITC for flow cytometry detection. (E) Quantitative analysis of (D). Data was expressed as mean ± SEM. *p < 0.05, **p < 0.01. (F) HCT8 cells were transfected with siNC and siBiP then treated with 30 μM LMPt for indicated time and endoplasmic reticulum stress and apoptosis related proteins were detected.

Then we investigated ER damage‐associated molecular pathways and its contribution to LMPt's anti‐cancer effect. Usually, ER stress would be activated in response to ER damage or dysfunction [14], and the ER stress‐mediating pathways include Bip–PERK–eIF2α–ATF4 and ATF6 pathways. Only when the damage is severe, these response pathways would be activated continuously to trigger pro‐apoptosis protein expression (CHOP, ATF4, cleaved‐ATF6) to induce apoptosis [15]. To further evaluate whether ER damage was also participating in the LMPt anti‐colorectal cancer effect, we detected ER stress pathways. Western blot results showed that LMPt activated the Bip–PERK–eIF2α–ATF4 pathway, and the activation was in a time‐dependent manner (Figure 2A), displaying an intensive and persistent response effect to ER damage.

Excessive endoplasmic reticulum stress activates the ATF4 pro‐apoptotic pathway to activate pro‐apoptotic proteins such as Bax and Puma [16]. Based on the above theories, we detected the pro‐apoptotic proteins Bax and Puma, anti‐apoptotic proteins Bcl‐2, Mal‐1 and Bcl‐xL (Figure 2B), and apoptotic effector protein (Figure 2C). The results showed that LMPt induced the expression of pro‐apoptotic protein and apoptotic effector protein, while the expression of anti‐apoptotic protein was significantly reduced. Subsequently, flow cytometry was used to further verify the apoptosis inducement (Figure 2D,E), indicating that LMPt induced ER stress and subsequent apoptosis. To further ascertain ER stress‐induced apoptosis, we knocked down Bip for the apoptosis detection and found the absence of Bip prevented LMPt's anti‐cancer action, discovering that with the entrance of LMPt into ER, the ER was damaged and ER stress was initiated to activate the Bip–PERK–eIF2α–ATF4 axis to trigger apoptosis to exhibit LMPt's anti‐colorectal action (Figure 2F).

## 2 Discussion

MPt is a third‐generation platinum; its water solubility is poor [17, 18, 19]. We prepared MPt into liposomes to form LMPt, with prior water solubility and significant pharmacological activity.

Our study revealed that LMPt effectively killed colorectal cancer cells. Since nanoparticles have a unique endocytosis pathway, we further explored the cellular entry pathway of LPMt. The endocytosis mechanisms of cells mainly include phagocytosis and pinocytosis, and pinocytosis can be divided into macropinocytosis and receptor‐mediated endocytosis. Receptor‐mediated endocytosis can be divided into clathrin‐mediated endocytosis, caveolin‐mediated endocytosis, and so on. We introduced different endocytosis pathway inhibitors to explore the potential mechanism of LMPt. The results showed that LPMt mainly passes through the Cav‐1 protein‐mediated endocytosis pathway into cells. In this process, after entering the cell, LMPt first enters and fuses with endosomes to form primary endosomes. The primary endosomes fuse with lysosomes during movement and are finally transferred to mitochondria. We speculate that this may be due to its unique lipid structure, which is similar to organelle membranes, so that it can be packaged and endocytosed, and finally reach the mitochondria to exert effects.

LMPt may induce cell death in colorectal cancer cells by triggering apoptosis and autophagy. In studying its mechanism of action, we found that after entering cells, LMPt can act on the endoplasmic reticulum, causing endoplasmic reticulum damage and inducing endoplasmic reticulum stress mediated by the Bip–PERK–eIF2α–ATF4 signalling pathway. This subsequently induces the expression of ATF4 and Bax, leads to changes in mitochondrial permeability, and ultimately induces mitochondrial apoptosis. In addition, LMPt can also be localised in mitochondria and cause mitochondrial damage, which is manifested as damage to the mitochondrial network structure; it increases ubiquitination of mitochondria and activates mitophagy. This mechanism is different from traditional platinum‐based anti‐tumour drugs. Most platinum‐based drugs mainly cross‐link with cellular DNA to form Pt‐DNA. Our research results have discovered possible potential targets for platinum anti‐tumour drugs and provide new ideas for the development and utilisation of this type of drugs. Similar to other anti‐cancer drugs, long‐term administration of LMPt also might occur drug resistance and if these events appear, the RNA‐seq or other analyses of drug‐resistance tumours could be carried out and some supplemental therapy might be beneficial to the overcoming of resistance. Meanwhile, for the future application of LMPt, patients with low caveolin‐1 might not be the optional beneficences and patient selection based on caveolin‐1 level is essential. In short, LMPt's low toxicity, high efficiency and unique anti‐cancer mechanism make it a promising drug for the treatment of colorectal cancer.

## Author Contributions

Wuli Zhao, Rongguang Shao and Guimin Xia designed and edited the manuscript; Cong Zhao, Yuhan Qiu, Xiaowei Wang, Mengyan Wang and Li Liu finished assays and wrote the manuscript.

## Funding

This work was supported by the Chinese Academy of Medical Sciences Innovation Fund for Medical Sciences (2023‐I2M‐2‐001, 2021‐I2M‐1‐030) and Beijing Natural Science Foundation (7254505).

## Conflicts of Interest

The authors declare no conflicts of interest.

## Supporting information


**Figure S1:** Superior anti‐colorectal cancer activity is observed in LMPt‐treated colorectal cancer. (A) The structure of miriplatin and LMPt. (B) HCT8 and HT29 cells were treated with 0.34 or 3.4 μM MPt for 48 h, then the MTT assay was used to detect cell viability. (C) Colorectal cancer cells HCT8 and HT29 were treated with 0, 3.75, 7.5, 15, 30 and 60 μM LMPt for 48 h, and cell survival was detected by MTT assay. A dose‐dependent curve was plotted by GraphPad Prism5 software. (D) Vitality of HCT8 and HT29 cells was detected by MTT assay after treatment with 2 μM LMPt for 0, 6, 12, 24 and 48 h. A time‐dependent curve was plotted. (E) The morphology of HCT8 and HT29 cells was observed after treatment with 0, 3, 10 and 30 μM LMPt. Scale bar, 10 μm. (F) HCT8 and HT29 cells were treated with 0, 3, 10 and 30 μM LMPt for 24 h, and cell proliferation was detected with EdU assay. Scale bar, 20 μm. (G) Quantitative analysis of (F). (H) HCT8 and HT29 cells were seeded in six‐well plates at the density of 1 × 10^3^ cells per well. After 24 h, various concentrations of LMPt were added and continued to incubate for 7 days for colony formation detection. Scale bar, 1 cm. (I) Quantitative analysis of (H). Colony formation rate = (numbers of colonies/numbers of seeded cells) × 100%. All the data were expressed as mean ± SEM (*n* = 3). *p < 0.05, **p < 0.01, ***p < 0.001, compared with control.
**Figure S2:** LMPt mainly locates in mitochondria and endoplasmic reticulum (ER) followed by preliminary cellular treatment. HCT8 cells were transfected with GFP‐labelled proteins endosomes and lysosomes and then treated with LMPt for specific time, the co‐localisation of LMPt with endosomes (A) and lysosomes (B) were observed by fluorescence microscope. Scale bar, 20 μm. (C) HCT8 and HT29 cells were treated with 30 μM LMPt for 24 h and the amount of platinum in mitochondria, endoplasmic reticulum and genomic DNA were determined by ICP–MS. (D) HCT8 cells were treated with 30 μM LMPt for 12 h and transmission electron microscopy was used to observe the mitochondrial morphology. Scale bar, 1 μm. (E) The mitochondria of HCT8 cells treated with 30 μM LMPt for 24 h were labelled with MitoTracker and representative images were acquired with a fluorescence microscope. (F) Quantitative analysis of (E). Mitochondrial network morphology was analysed by ImageJ. Scale bar, 5 μm. (G) HCT8 and HT29 cells were treated with 0, 10 or 30 μM LMPt for 24 h, and then stained with JC‐1 and mitochondrial membrane potential was observed by fluorescence microscope. Scale bar, 50 μm. (H) Observation of morphological changes of endoplasmic reticulum in HCT8 cells after treatment with 30 μM LMPt under a fluorescence microscope. The ER–GFP plasmid that marks the endoplasmic reticulum lumen and the ER‐Sec61β plasmid that marks the endoplasmic reticulum membrane were transfected into HCT8 cells to establish a cell line stably expressing ER–GFP and ER‐Sec61β. Scale bar, 20 μm.
**Figure S3:** LMPt induces mitophagy and endoplasmic reticulum stress‐mediated apoptosis. (A) HCT8 cells were treated with 30 μM LMPt for 24 h and RNA was extracted for RNA sequencing. KEGG pathway enrichment analysis of differentially expressed genes between control group and LMPt treated group. (B) HCT8 and HT29 (C) cells were treated with Z‐VAD, 3‐MA and Nec‐1 prior to the addition of LMPt, and the cell survival was examined. Data was expressed as mean ± SEM. ns, no significant. *p < 0.05, **p < 0.01. (D) Observation of the effect of LMPt on the co‐localisation of lysosome and mitochondria in HCT8 cells under fluorescence microscope. Scale bar, 10 μm. (E) The expression levels of mitophagy related proteins in HCT8 and HT29 cells treated with LMPt were detected by immunoblot. HCT8 and HT29 cells were treated with LMPt for 24 h, and cytoplasm and mitochondria were isolated from the harvested cells. LC3 and ubiquitin (F) were determined by immunoblot.

## Data Availability

The data that support the findings of this study are available on request from the corresponding author. The data are not publicly available due to privacy or ethical restrictions.
