# Supplementary material for: Mitophagy and Bip–PERK–eIF2α–ATF4 Axis‐Mediated ER Stress Mediate Miriplatin‐Loaded Liposome's Anti‐Colorectal Cancer Action
Source: Cell Prolif. 2025 Dec 19;59(3):e70153. doi: 10.1111/cpr.70153 (PMC12961539; doi:10.1111/cpr.70153)
Supplement: Supplementary file 1 — Figure S1: Superior anti‐colorectal cancer activity is observed in LMPt‐treated colorectal cancer. (A) The structure of miriplatin and LMPt. (B) HCT8 and HT29 cells were treated with 0.34 or 3.4 μM MPt for 48 h, then the MTT assay was used to detect cell viability. (C) Colorectal cancer cells HCT8 and HT29 were treated with 0, 3.75, 7.5, 15, 30 and 60 μM LMPt for 48 h, and cell survival was detected by MTT assay. A dose‐dependent curve was plotted by GraphPad Prism5 software. (D) Vitality of HCT8 and HT29 cells was detected by MTT assay after treatment with 2 μM LMPt for 0, 6, 12, 24 and 48 h. A time‐dependent curve was plotted. (E) The morphology of HCT8 and HT29 cells was observed after treatment with 0, 3, 10 and 30 μM LMPt. Scale bar, 10 μm. (F) HCT8 and HT29 cells were treated with 0, 3, 10 and 30 μM LMPt for 24 h, and cell proliferation was detected with EdU assay. Scale bar, 20 μm. (G) Quantitative analysis of (F). (H) HCT8 and HT29 cells were seeded in six‐well plates at the density of 1 × 10^3^ cells per well. After 24 h, various concentrations of LMPt were added and continued to incubate for 7 days for colony formation detection. Scale bar, 1 cm. (I) Quantitative analysis of (H). Colony formation rate = (numbers of colonies/numbers of seeded cells) × 100%. All the data were expressed as mean ± SEM (n = 3). *p < 0.05, **p < 0.01, ***p < 0.001, compared with control. Figure S2: LMPt mainly locates in mitochondria and endoplasmic reticulum (ER) followed by preliminary cellular treatment. HCT8 cells were transfected with GFP‐labelled proteins endosomes and lysosomes and then treated with LMPt for specific time, the co‐localisation of LMPt with endosomes (A) and lysosomes (B) were observed by fluorescence microscope. Scale bar, 20 μm. (C) HCT8 and HT29 cells were treated with 30 μM LMPt for 24 h and the amount of platinum in mitochondria, endoplasmic reticulum and genomic DNA were determined by ICP–MS. (D) HCT8 cells were treated with 30 μM LMPt for 12 h and tran [file CPR-59-e70153-s001.docx]

**Supplementary material**

**Material and methods**

**Cell culture**

Human colon cancer HCT8 and HT29 cell lines were purchased from the Institute of Basic Medical Sciences, Chinese Academy of Medical Sciences (Beijing, China). Cells are rapidly thawed in the 37°C water bath, HCT8 cells were grown in DMEM medium, HT29 cells were grown in DMEM/F12 medium. 10% Fetal bovine serum（FBS）and 1% Penicillin/Streptomycin (P/S) were added to the culture medium. Cell culture conditions were 37°C, 5% CO2. The DMEM, DMEM/F12 culture medium, 100 x P/S solution, PBS and 0.25% Trypsin-EDTA were purchased from MACGENE Company (Beijing, China). FBS was purchased from Gibco (Grand Island, NY, USA).

**MTT assay**

Cells in the logarithmic growth phase were counted, and seeded in a 96-well plate at a density of 5 × 10^3^ cells/well and cultured at 37°C, 5% CO_2_ for 24 h. The LMPt was diluted to the target concentration with medium and mixed evenly, and then treated with compound for 48 h. Discard the medium and add MTT 1mg/mL. After 4 h, 150 μL DMSO was added to each well and the absorbance value was detected by microplate reader. MTT reagent was purchased from GPC (Beijing, China).

**Colony formation assay**

Cells in the logarithmic growth phase were seeded in six-well plate at a density of 1 × 10^3^ cells/well, and LMPt was added after 24 h. Then continue to culture for 7 days, stop the culture when there are visible clones in the plate, wash with PBS, stain crystal violet for 15 min, take pictures and count after washing with distilled water, and calculate the cloning rate. Crystal violet dyeing solution was purchased from Beyotime Biotechnology (Beijing, China).

**1.4** **5-ethynyl-2'-deoxyuridine (EdU) assay**

Cells were seeded in 24-well plates with pre-placed cell crawlers, 4 × 10^4^ cells/well, and incubated at 37°C for 24 h. Next, cells are treated with different concentrations of drug and then incubated with EdU medium for 2 h. After washing with PBS, the cells were fixed with 4% paraformaldehyde, incubated with glycine solution for 5 min, and 0.5% TritonX-100 for 10 min. Next, the Apollo stain solution was incubated at room temperature for 30 min. After PBS washing, the crawler was inverted on the cationic anti-detachment slide, the nuclei were stained with DAPI, and the microscope was photographed for observation. The Cell-Light 5-ethynyl-2'-deoxyuridine (EdU) kit was purchased from RiboBio Company (Guangzhou, China).

**Drug uptake studies**

Logarithmic cells were seeded in 6-well plates at a density of 3 × 10^5^ cells/well and incubated at 37°C for 24 h. The drug with a red fluorescent label is loaded with fluorescence DiI and diluted with medium to target concentration for administration. Then removed the drug, washed by PBS, and fresh medium was added and observed under a fluorescence microscope. Alternatively, the cells after drug treatment are digested and collected, and the red fluorescence intensity is quantitatively detected using a flow cytometry after resuspension in PBS.

**Exploration of the endocytic mechanism of LMPt**

Cells were seeded in a 6-well plate at a density of 3 × 10^5^ cells/well, and incubated for 24 h. Next, the cells were pre-treated with various endocytosis inhibitors for 2 h, and the inhibitor concentrations were 10 μM genistein, 10 μM amiloride, 5 μg/mL chlorpromazine, 0.1 μg/mL cytochalasin B and 0.1 μg/mL colchicine. After 2 h, 10 μM LMPt were added and continued to culture for 24 h. According to the experimental methods, cell viability detection and intracellular platinum content detection experiments were carried out respectively.

**ICP-MS assay**

Control cells and drug-treated cells were collected into 1.5 mL centrifuge tubes at 1000 rpm for 5 min. Discard the supernatant, resuspend in PBS and perform cell counting, then centrifuge again and discard the supernatant to allow the cells to pellet the sample. 150 μL of nitric acid was added to each cell sample, nitrated overnight, and the sample was diluted 20 times with 0.2% dilute nitric acid before sample delivery for testing, and the dilution factor was adjusted according to the number of cells in the sample before detection. The intracellular mass of platinum element was detected by ICP-MS (ELAN DRC-II, PerkinElmer, USA).

**Mitochondrial extraction**

Cells from the control group and the drug-treated group were collected, and the supernatant was discarded after centrifugation at 850 g for 2 min. Mitochondrial Isolation Reagent A with 1% PMSF was added, vortex for 5 sec, and let stand on ice for 2 min. Add Mitochondrial Isolation Reagent B and vortex at the highest speed for 5 sec. Incubate on ice for 5 min with vortex per minute at the highest speed. Next, add Mitochondrial Isolation Reagent C with 1% PMSF to each tube and mix well. After centrifugation, transfer the supernatant to a new centrifuge tube and centrifuge again. Mitochondrial separation reagent C was added to the pellet and centrifuged for 5 min, and the supernatant was discarded. Precipitate into isolated mitochondria. Mitochondria isolation kit for cultured cells was purchased from Thermo Scientific.

**Extraction of endoplasmic reticulum**

Cells from the control group and the drug treatment group were collected, centrifuged at 600 g for 5 min, the supernatant was discarded, washed with PBS, and centrifuged again. Discard the supernatant and measure the precipitate volume. Add 3 volumes of hypotonic extraction buffer containing 1% PMSF to the pellet, resuspend the pellet, and incubate for 20 min to swell the cells. At 4°C, the rotation speed was 600 g, centrifugation for 5min, the supernatant was discarded, and the precipitate volume was measured. Add 2 volumes of isotonic extraction buffer containing 1% PMSF to the pellet, resuspend the pellet, and transfer to a 1 mL syringe. Aspirate several times repeatedly with a homogenization tube to obtain homogenized cell fluid. Centrifuge at 1 × 10^3^ g for 10 min at 4 °C, carefully aspirate the top lipid layer, and transfer the supernatant to a new centrifuge tube. Continue centrifugation, carefully aspirate the top lipid layer, transfer the supernatant to a new centrifuge tube, add CaCl_2_ solution to mix well, and incubate for 15 min. Centrifuge at 4 °C for 10 min to precipitate endoplasmic reticulum. The endoplasmic reticulum isolation kit was purchased from Sigma Aldrich.

**Transmission electron microscopy detection**

After treated HCT8 cells with 30 μM LMPt for 12 h, collected the cells and centrifuge at 1 × 10^3^ g for 5 min. Washed with PBS and discarded the supernatant. Then 2.5% glutaraldehyde was diluted 10 times with PBS. Slowly added the prepared fixative into the cell pellet to form aggregates of fixed cells. Fixed the cells at 4°C overnight, and then dehydrated with increasing concentrations of ethanol (50%, 60%, 70%, 80%, 90%, and 100%). Next, the cells were stained with 2% uranyl acetate overnight at room temperature and then embedded in Epon. Then samples were sectioned into 60-nm-thick slices and observed by a JEM-1400 Plus transmission electron microscopy system (JEOL, Japan).

**Extraction of genomic DNA**

Collected LMPt-treated cells in centrifuge tubes, then added 100 μL LB2 solution to each tube, resuspended the cells and mixed well. In order to remove RNA, added 20 μL RNaseA to each tube and incubated for 2 min at room temperature. Added 20 μL ProteinaseK and incubated at room temperature for 2 min. Added 500 μL BB2 solution, and vortexed immediately for 5 sec, and then incubated for 10 min. Added the whole solution to the spin column, centrifuged at 1.2 × 10^4^ g for 30 sec, and discarded the effluent.
 Added CB2 and centrifuged for 30 sec, then washed with WB2. Placed the spin column in a clean centrifuge tube, added 100 μL pre-warmed 65°C EB, centrifuged at 2 × 104 g for 1 min, and eluted the DNA. The eluted DNA can be stored at -20°C. The Genomic DNA extraction kit was purchased from TransGen Biotech.

**RNA sequencing (RNA-seq)**

The cells were treated with 30 μM LMPt for 24 h, and then added trizol to disrupt the cells and collected into the centrifuge tubes. Then let stand at room temperature for 5 min to allow the cells to fully lyse, and then added 200 μL chloroform to the lysate of each sample, shaked vigorously for 15 sec, and let stand at room temperature for 15 min. Centrifuged at 4 °C, 1.2 × 10^4^ rpm for 15 min and carefully pipetted the upper aqueous phase to a new tube. Next, added 600μL isopropanol to each tube, inverted and then let it stand at room temperature for 10min. Then centrifuged and a white precipitate at the bottom of the tube can be observed after centrifugation, which is RNA. Carefully aspirate the supernatant and remove it as much as possible, dissolving the RNA using RNase-free water pre-warmed at 65 °C in advance.
Determined the total RNA concentration and purity of the extracted with a NanoDrop 2000 then sent it to the Beijing Genomics Institute (BGI, Shenzhen, China) for RNA extraction, complementary DNA (cDNA) library construction and sequencing.

**Apoptosis assays**

Cells were treated with different concentrations of LMPt for 48 h, then collected into centrifuge tubes, and washed in PBS. Add 195 μL of Annexin V-FITC binding solution to resuspend the cells, add 5 μL of Annexin V-FITC to mix well, then add 10μL of PI staining solution to mix, incubate in the dark for 20 min, filter the sample and then detect by flow cytometry. Apoptosis detection kit was purchased from Beyotime Biotechnology (Beijing, China).

**Mitochondrial membrane potential changes detection**

The cells were treated with different concentrations of LMPt for 24 h, and JC-1 staining solution was added and incubated in the incubator for 20 min. Discard the staining solution and gently wash the cells 2 times with culture medium. Add fresh complete medium and observe under a fluorescence microscope. Mitochondria staining kit for mitochondrial potential changes detection was purchased from Sigma Aldrich.

**Plasmids and siRNA transfection**

Caveolin-1 siRNA was purchased from RiboBio Company (Guangzhou, China). Seeded 3 × 10^5^ cells/well in the 6-well plate for transient transfection of tumor cells. Then incubated for 24 h, replaced the serum-free medium and the cells were transfected with Lipofectamine 2000 Transfection reagent or Lipofectamine RNAiMAX Transfection reagent in opti-MEM. Transfection reagent was purchased from Invitrogen (Thermo Fisher Scientific, USA). Opti-MEM was purchased from Gibco (Grand Island, USA).

**Western blot**

Collected the cells, lysed them with RIPA containing 1% PMSF for 30 min at 4°C, collected the supernatant after centrifugation, and performed quantification by BCA method. Then boiled at 100°C for 10 min for denaturation. Next, performed SDS-PAGE gel electrophoresis and transfered to membrane. After blocking with 5% skim milk for 2 h, the protein primary antibody was incubated overnight at 4°C. Protein secondary antibodies were incubated at room temperature for 2 h. ECL luminescent liquid was used for exposure processing. RIPA、PMSF and BCA kit were purchased from Beyotime Biotechnology (Beijing, China). PVDF membrane and ECL luminescent liquid were purchased from Merck Millipore Ltd. (Tullagreen, Carrigtwohill, Ireland). The antibodies used in the experiment were as follows: anti-GAPDH Rabbit mAb (Cell Signaling Technology, #5174), anti-eIF2α Rabbit mAb (Cell Signaling Technology, #5324), anti-ATF4 Rabbit mAb (Beyotime Biotechnology, AF2560), anti-BiP Rabbit mAb (Beyotime Biotechnology, AB310), anti-P-eIF2α Rabbit mAb (Beyotime Biotechnology, AF1237), anti-Puma Rabbit mAb, anti-Bax Rabbit mAb (Pro-Apoptosis Bcl-2 Family Antibody Sampler Kit, Cell Signaling Technology, #9942), anti-Bcl-xL Rabbit mAb, anti-Bcl-2 Rabbit mAb, anti-Mcl-1 Rabbit mAb (Pro-Survival Bcl-2 Family Antibody Sampler Kit, Cell Signaling Technology, #9941), anti-Cleaved PARP Rabbit mAb, anti-Cleaved Caspase3 Rabbit mAb, anti-Cleaved Caspase9 Rabbit mAb (Apoptosis Antibody Sampler Kit, Cell Signaling Technology, #9915), anti-COX IV Rabbit mAb (Cell Signaling Technology, #4850), anti-LC3 Rabbit mAb (Proteintech, 14600-1-AP), anti-PINK1 Rabbit mAb (Cell Signaling Technology, #6946), anti-Ubiquitin Rabbit mAb (Cell Signaling Technology, #3933).

The graphical abstract of this study was drawn using Generic Diagramming Platform (GDP)^[20]^.

**Statistical analysis**

Statistical results were presented as the mean standard deviation (SD) of at least three independent experiments. Statistical significance was performed using Graphpad Prism software 7.0 version (GraphPad Software company, San Diego, CA, USA). Multiple group comparison was performed using ordinary one-way ANOVA with Dunnett’s multiple comparison tests. Two-group comparison was performed using two-tailed Student’s t test. P < 0.05 was regarded as statistical significance, symbolized by *P < 0.05, **P < 0.01, ***P < 0.001 and not significant (ns).

**Supplementary figure**


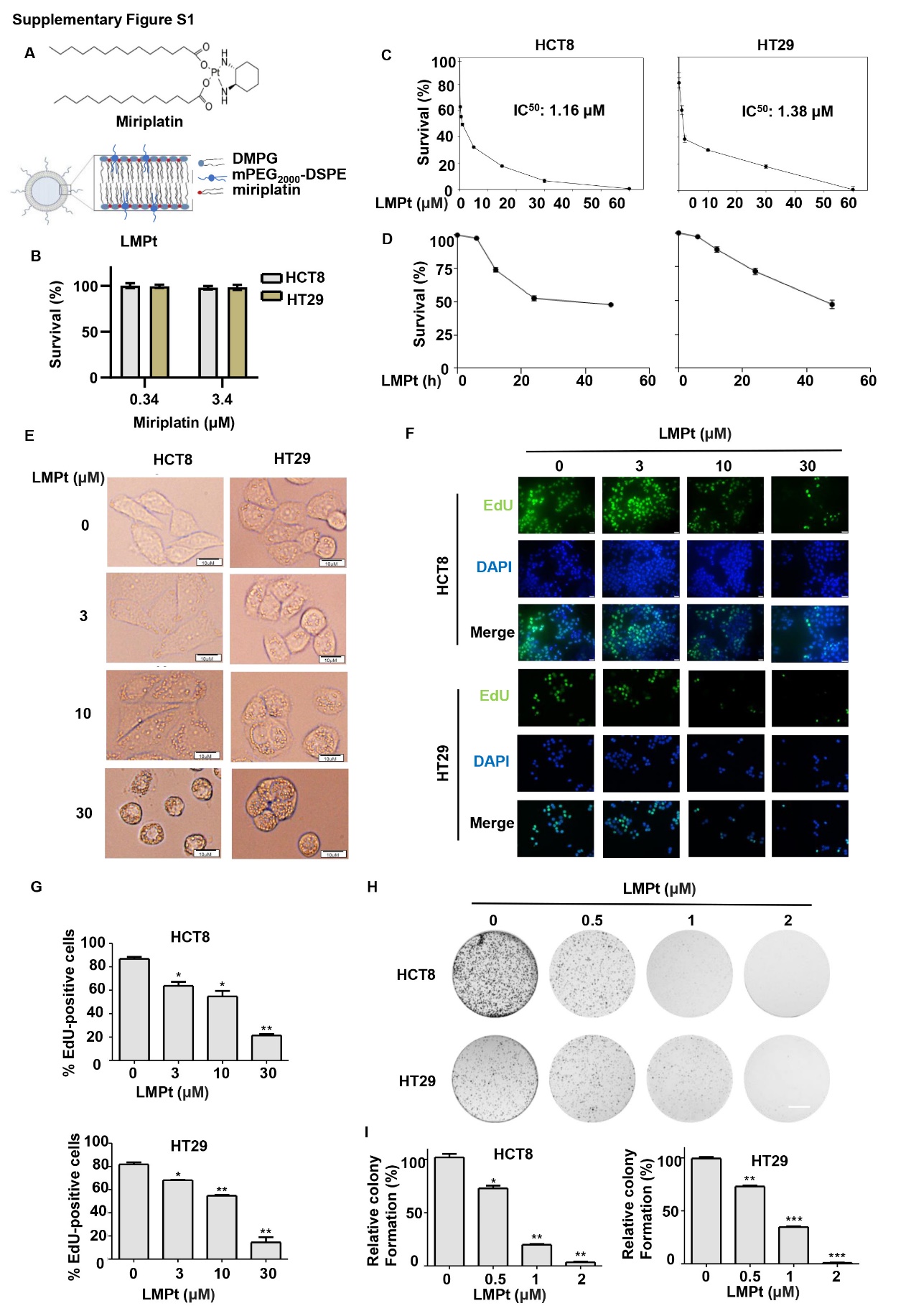


**
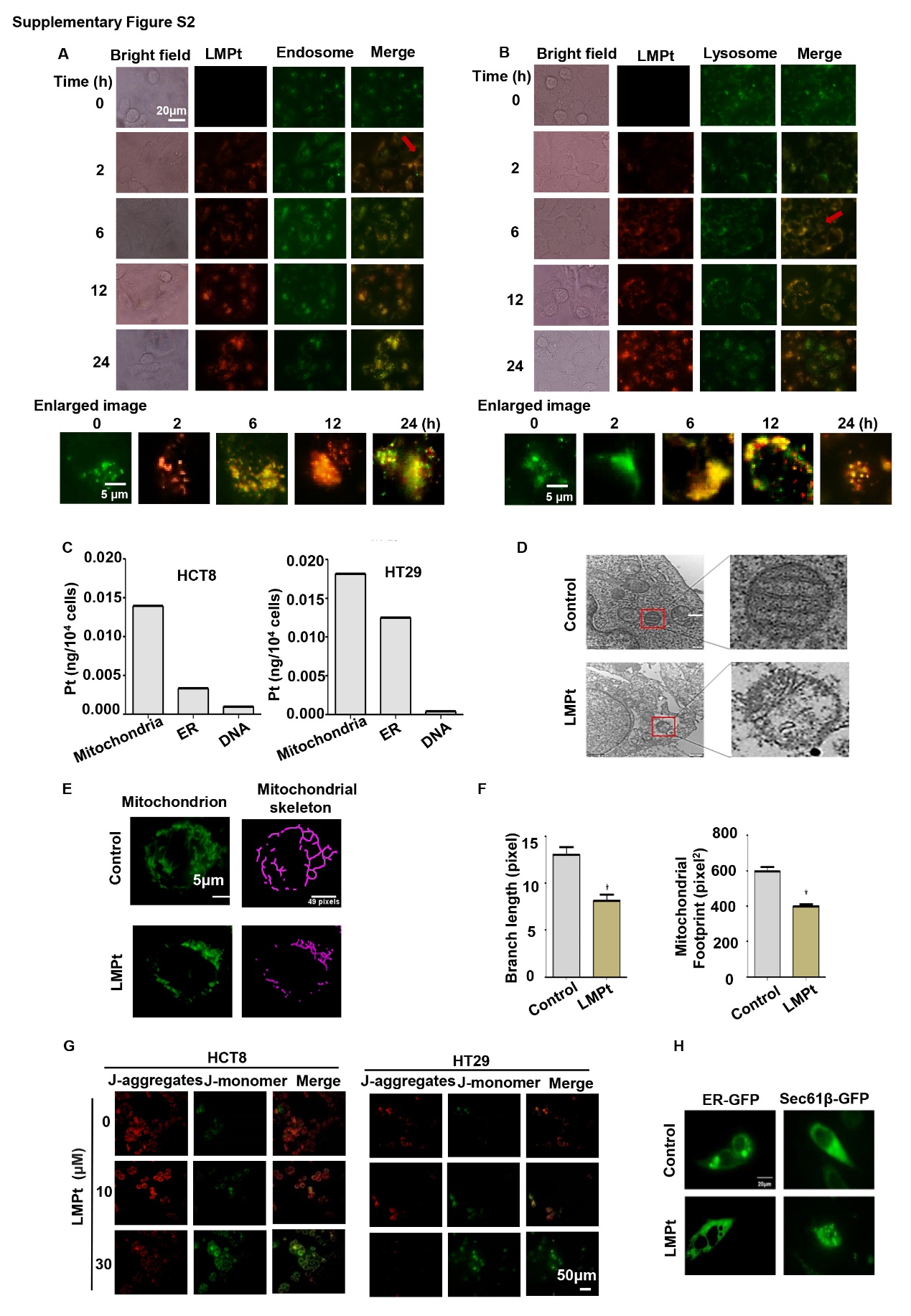
**

**
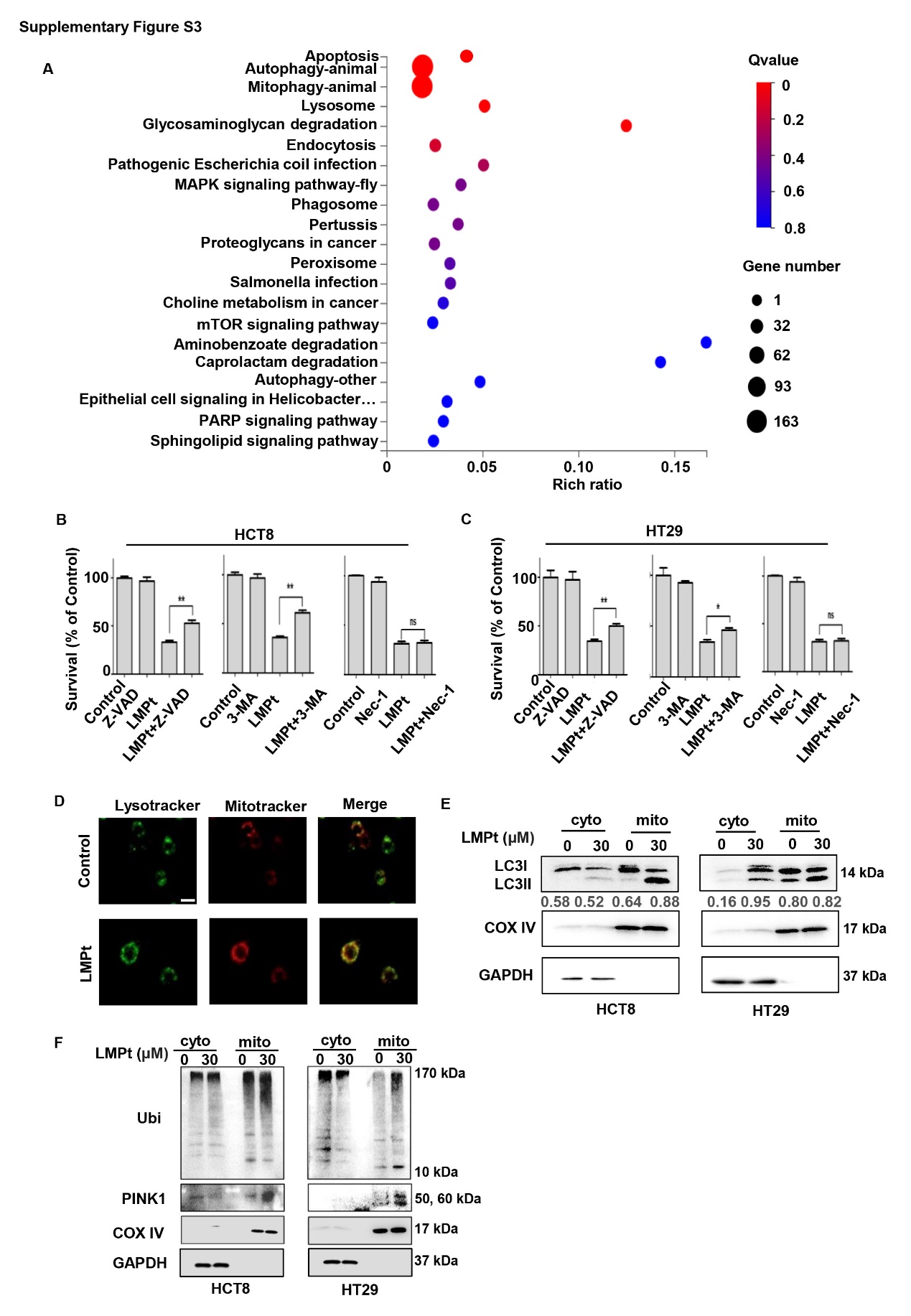
**

**Legends:**

**Supplementary figure 1. Superior anti-colorectal cancer activity is observed in LMPt-treated colorectal cancer.**

(A) The structure of miriplatin and LMPt. (B) HCT8 and HT29 cells were treated with 0.34 μM or 3.4 μM MPt for 48 h, then the MTT assay was used to detect cell viability. (C) Colorectal cancer cells HCT8 and HT29 were treated with 0 μM, 3.75 μM, 7.5 μM, 15 μM, 30 μM and 60 μM LMPt for 48 h, and cell survival was detected by MTT assay. A dose-dependent curve was plotted by GraphPad Prism5 software. (D) Vitality of HCT8 and HT29 cells was detected by MTT assay after treatment with 2 μM LMPt for 0 h, 6 h, 12 h, 24 h, 48 h. A time-dependent curve was plotted. (E) The morphology of HCT8 and HT29 cells was observed after treatment with 0 μM, 3 μM, 10 μM and 30 μM LMPt. Scale bar, 10 μm. (F) HCT8 and HT29 cells were treated with 0 μM, 3 μM, 10 μM and 30 μM LMPt for 24 h, and cell proliferation was detected with EdU assay. Scale bar, 20 μm. (G) Quantitative analysis of Fig.S1F. (H) HCT8 and HT29 cells were seeded in 6-well plates at the density of 1 × 10^3^ cells per well. After 24 h, various concentrations of LMPt were added and continued to incubate for 7 days for colony formation detection. Scale bar, 1cm. (I) Quantitative analysis of Fig.S1H. Colony formation rate = (numbers of colonies / numbers of seeded cells) × 100%. All the Data were expressed as Mean ± SEM (n=3). **p* < 0.05, ***p* < 0.01, ****p* < 0.001, compared with control.

**Supplementary figure 2. LMPt mainly locates in mitochondria and endoplasmic reticulum (ER) followed by preliminary cellular treatment.**

HCT8 cells were transfected with GFP-labeled proteins endosomes and lysosomes and then treated with LMPt for specific time, the co-localization of LMPt with endosomes (A) and lysosomes (B) were observed by fluorescence microscope. Scale bar, 20 μm. (C) HCT8 and HT29 cells were treated with 30 μM LMPt for 24 h and the amount of platinum in mitochondria, endoplasmic reticulum, and genomic DNA were determined by ICP-MS. (D) HCT8 cells were treated with 30 μM LMPt for 12 h and transmission electron microscopy was used to observe the mitochondrial morphology. Scale bar, 1μm. (E) The mitochondria of HCT8 cells treated with 30 μM LMPt for 24 h were labeled with MitoTracker and representative images were acquired with a fluorescence microscope. (F) Quantitative analysis of Fig.S2E. Mitochondrial network morphology was analyzed by ImageJ. Scale bar, 5 μm. (G) HCT8 and HT29 cells were treated with 0 μM, 10 μM or 30 μM LMPt for 24 h, and then stained with JC-1 and mitochondrial membrane potential was observed by fluorescence microscope. Scale bar, 50 μm. (H) Observation of morphological changes of endoplasmic reticulum in HCT8 cells after treatment with 30 μM LMPt under a fluorescence microscope. The ER-GFP plasmid that marks the endoplasmic reticulum lumen and the ER-Sec61β plasmid that marks the endoplasmic reticulum membrane were transfected into HCT8 cells to establish a cell line stably expressing ER-GFP and ER-Sec61β. Scale bar, 20 μm.

**Supplementary figure 3. LMPt induces mitophagy and endoplasmic reticulum stress-mediated apoptosis.**

(A) HCT8 cells were treated with 30 μM LMPt for 24 h and RNA was extracted for RNA sequencing. KEGG pathway enrichment analysis of differentially expressed genes between control group and LMPt treated group. (B) HCT8 and HT29 (C) cells were treated with Z-VAD, 3-MA and Nec-1 prior to the addition of LMPt, and the cell survival was examined. Data was expressed as Mean ± SEM. ns, no significant. **p* < 0.05, ***p* < 0.01. (D) Observation of the effect of LMPt on the co-localization of lysosome and mitochondria in HCT8 cells under fluorescence microscope. Scale bar, 10 μm. (E) The expression levels of mitophagy related proteins in HCT8 and HT29 cells treated with LMPt were detected by immunoblot. HCT8 and HT29 cells were treated with LMPt for 24 h, and cytoplasm and mitochondria were isolated from the harvested cells. LC3 and ubiquitin (F) were determined by immunoblot.
